# Phylogenomics of the *Olea europaea* complex using 15 whole genomes supports recurrent genetic admixture together with differentiation into seven subspecies

**DOI:** 10.1186/s12915-023-01583-5

**Published:** 2023-04-17

**Authors:** Irene Julca, Pablo Vargas, Toni Gabaldón

**Affiliations:** 1 Barcelona Supercomputing Centre (BSC-CNS), Plaça Eusebi Güell, 1-3, 08034 Barcelona, Spain; 2 Institute for Research in Biomedicine (IRB Barcelona), The Barcelona Institute of Science and Technology, Baldiri Reixac, 10, 08028 Barcelona, Spain; 3 School of Biological Sciences, Nanyang Technological University, 60 Nanyang Drive, Singapore, 637551 Singapore; 4 Department of Biodiversity and Conservation, Real Jardín Botánico de Madrid, Calle Claudio Moyano 1, 28014 Madrid, Spain; 5 Catalan Institution for Research and Advanced Studies (ICREA), Barcelona, Spain; 6 CIBER de Enfermedades Infecciosas, Instituto de Salud Carlos III, Madrid, Spain

**Keywords:** Macaronesian islands, Olive (Olea europaea), Phylogenomics, Polyploidy, Subspecies

## Abstract

**Background:**

The last taxonomic account of *Olea* recognises six subspecies within *Olea europaea* L., including the Mediterranean olive tree (subsp. *europaea*) and five other subspecies (*laperrinei*, *guanchica*, *maroccana*, *cerasiformis*, and *cuspidata*) distributed across the Old World, including Macaronesian islands. The evolutionary history of this monophyletic group (*O. europaea* complex) has revealed a reticulated scenario involving hybridization and polyploidization events, leading to the presence of a polyploid series associated with the subspecies. However, how the polyploids originated, and how the different subspecies contributed to the domestication of the cultivated olive are questions still debated. Tracing the recent evolution and genetic diversification of the species is key for the management and preservation of its genetic resources. To study the recent history of the *O. europaea* complex, we compared newly sequenced and available genomes for 27 individuals representing the six subspecies.

**Results:**

Our results show discordance between current subspecies distributions and phylogenomic patterns, which support intricate biogeographic patterns. The subspecies *guanchica*, restricted to the Canary Islands, is closely related to subsp. *europaea*, and shows a high genetic diversity. The subsp. *laperrinei*, restricted now to high mountains of the Sahara desert, and the Canarian subsp. *guanchica* contributed to the formation of the allotetraploid subsp. *cerasiformis* (Madeira islands) and the allohexaploid subsp. *maroccana* (western Sahara region). Our phylogenomic data support the recognition of one more taxon (subsp. *ferruginea*) for the Asian populations, which is clearly segregated from the African subsp. *cuspidata*.

**Conclusions:**

In sum, the *O. europaea* complex underwent several processes of hybridization, polyploidy, and geographical isolation resulting in seven independent lineages with certain morphological traits recognised into subspecies.

**Supplementary Information:**

The online version contains supplementary material available at 10.1186/s12915-023-01583-5.

## Background

**Fig. 1:**
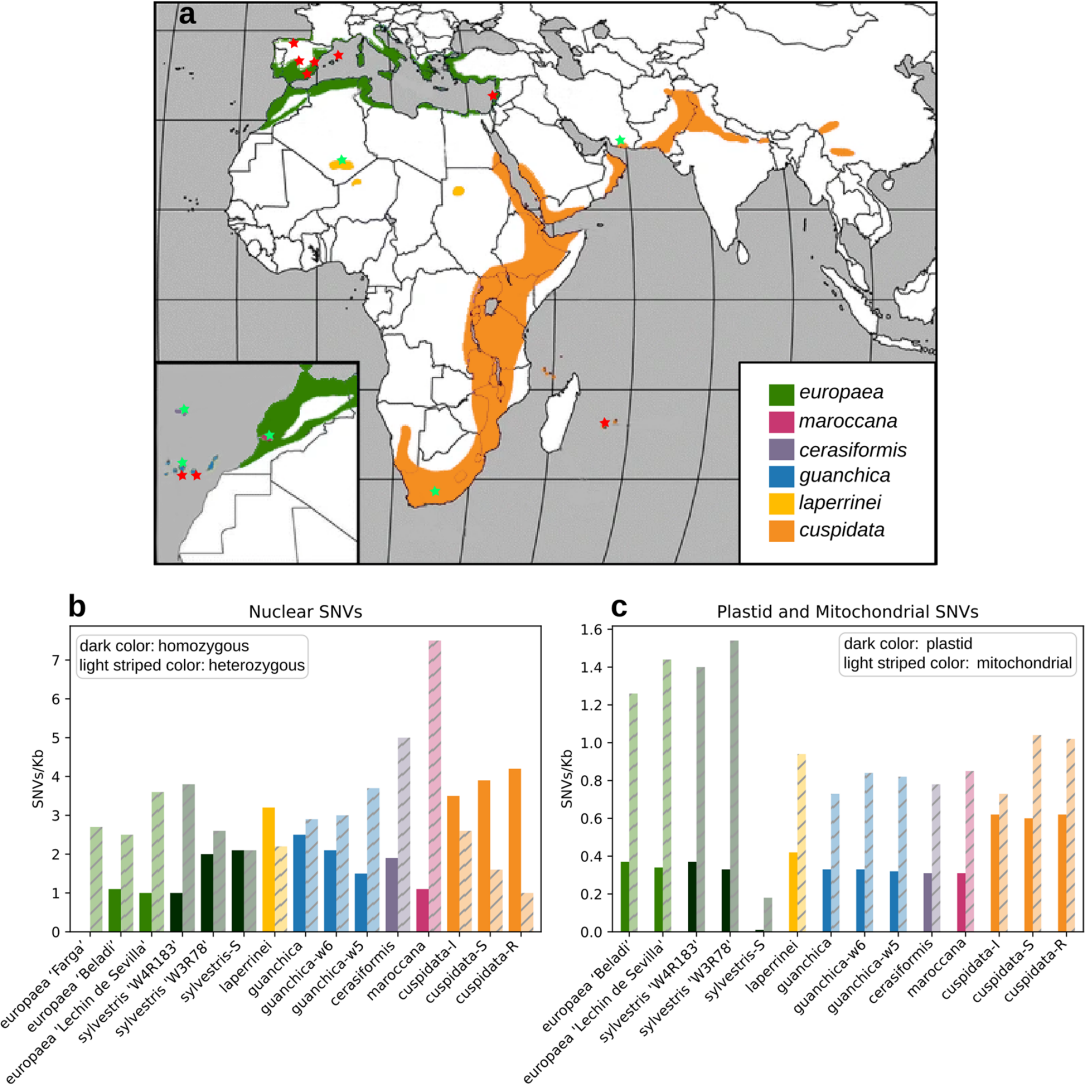
*Olea europaea* sampling and SNV densities (SNVs/Kb). **a** Geographical distribution of the six *O. europaea* subspecies (modified from [8]). Samples sequenced in this project are marked with green stars and other whole-genome sequences with red stars. Colours show the distribution of each subspecies. **b** SNVs/Kb for homozygous (darker colours) and heterozygous (lighter striped colours) SNVs in the nuclear genome. **c** SNV densities for the plastid (dark colour) and mitochondrial (light striped colour) genomes

The *Olea europaea* complex [1] includes the wild and cultivated Mediterranean olives (*O. europaea* subspecies *europaea*), and five non-Mediterranean subspecies (Fig. 1a): subsp. *laperrinei* distributed in Saharan massifs; subsp. *cuspidata*, with a broad distribution ranging from South Africa to southern Egypt and from Arabia to northern India and southwestern China; subsp. *guanchica* endemic to the Canary Islands; subsp. *maroccana* in the Agadir Mountains (western Sahara region); and subsp. *cerasiformis* in the Madeira islands [2, 3]*.* The subsp. *europaea* is further subdivided into two taxonomic varieties: var. *sylvestris*, also named oleaster or wild olive, which comprises the wild forms of the olive tree, and var. *europaea*, which encompasses around 1,000 cultivated forms [2, 4]. Recent analyses of the first complete genome of *O. europaea* have uncovered several ancient polyploidization events in the lineage leading to this species, of which two were described as allopolyploidization events [5, 6]. In addition to these ancient events, earlier work had described hybridization processes within the *O. europaea* complex [4, 7, 8] and had identified the presence of a polyploid series, including some derived diploid subspecies (*europaea*, *laperrinei*, *cuspidata*, *guanchica*) with 2n = 46, the tetraploid subsp. *cerasiformis*, and the hexaploid subsp. *maroccana* [1, 9]. However, these studies are limited because of the use of a low sample size in terms of the number of genetic markers. Therefore, the question remains as to how the polyploids originated, and how the different subspecies contributed to the domestication of the cultivated olive. In addition, a recent genomic analysis of cultivars and wild relatives has identified common introgression and hybridization [10], but the extent to which such processes have operated in the earlier diversification of the subspecies remains to be disclosed. Access to genome sequences of additional subspecies of the *O. europaea* complex is necessary to understand the recent evolution of the species and to assess the genomic aftermath of past processes of isolation, hybridization and whole-genome duplication. To infer the recent evolution of the *O. europaea* complex, we whole sequenced one individual of each of the following subspecies: *cerasiformis*, *maroccana*, *guanchica*, *laperrinei*, and two individuals of the subspecies *cuspidata* from two continents (Africa, Asia). We integrated these sequenced individuals with available genome sequences of the subspecies *europaea*, *guanchica*, and *cuspidata*. A comparative analysis of a total of 27 olive genomes is addressed to shed further light on the evolutionary history of the *O. europaea* complex.

## Results

### Patterns of genetic polymorphism in *O. europaea*

To study patterns of genetic polymorphism within *Olea europaea*, we sequenced six individuals of the five subspecies of the *O. europaea* complex and retrieved available sequences from ENA and NCBI databases: nuclear genome sequences for nine individuals of subspp. *guanchica*, *europaea* and *cuspidata*, and 12 plastid sequences from all six subspecies. These samples represent the current geographic distribution of the species (Fig. 1a, Table 1). Using a read mapping strategy (see Methods), we called single nucleotide variants (SNVs), which resulted in a total of 27,877,747 nuclear, 3,385 mitochondrial, and 238 plastidial polymorphic positions. The number of identified homozygous nuclear SNVs per individual shows that subsp. *cuspidata* has the largest numbers (~ 3.9 SNVs/Kb), followed by subsp. *laperrinei* (3.2 SNVs/Kb) (Fig. 1b). The other subspecies have a similar amount of homozygous SNVs with respect to the reference. Interestingly, the two polyploid subspecies [9] *maroccana* (6x), and *cerasiformis* (4x) showed significantly higher amounts of heterozygous SNVs as compared to the other individuals, suggesting hybridization events (including introgression) have been involved in the origin of their polyploidy. The analysis of plastid SNVs (Fig. 1c, darker colours) resulted in a similar ordering of genetic distances to the reference of the different subspecies (*cuspidata* > *laperrinei* > other). However, for mitochondrial SNVs (Fig. 1c, lighter striped colours) the subspecies *europaea* shows a higher number of SNVs than the other subspecies. The individual with the lowest number of plastid and mitochondrial polymorphic positions is the subsp. *europaea* var. *sylvestris*—S, as already noted in our previous study [10].

**Table 1:** Twenty-seven genomes of *O. europaea* used in the analysis. Columns show taxon, geographical origin, plastid group [11], ploidy level, genomes analysed, and data source

Taxon (subspecies)	Geographic origin	Plastid group	Ploidy level	Genome analysed	Source
*europaea* var. *europaea* cv. ‘Farga’	Spain (Boadilla/La Senia)	E3.1	2x	nuclear, plastid, mitochondrial	ENA (PRJEB4992)
*europaea* var. *europaea* cv. 'Lechin de Sevilla'	Spain	E2.3	2x	nuclear, plastid, mitochondrial	ENA (PRJEB35540)
*europaea* var. *europaea* cv. ‘Beladi’	Lebanon	E1.1	2x	nuclear, plastid, mitochondrial	ENA (PRJEB35540)
*europaea* var. *sylvestris*	Spain (Pechón)	E3	2x	nuclear, plastid, mitochondrial	ENA (PRJEB35540)
*europaea* var. *sylvestris* 'W4R183'	Spain	E1	2x	nuclear, plastid, mitochondrial	NCBI (SRR9860503)
*europaea* var. *sylvestris* 'W3R78'	Spain (Menorca)	E2	2x	nuclear, plastid, mitochondrial	NCBI (SRR9860505)
*europaea* var. *sylvestris* 'Stavrovouni 11'	Cyprus	E1.4	2x	plastid	NCBI (HF558645)
*europaea* var. *sylvestris* 'Haut Atlas 1'	Morocco (High Atlas)	E2	2x	plastid	NCBI (NC_015401)
*europaea* var. *sylvestris* 'Gue de Constantine 20'	Algeria (Gue de Constantine)	E3	2x	plastid	NCBI (FN997651)
*europaea* var. *sylvestris* ‘Oeiras 1’	Portugal	E3.1	2x	plastid	NCBI (MG255763)
*europaea* var. *sylvestris* ‘Vallee du Fango 5’	France	E2.1	2x	plastid	NCBI (MG255762)
*maroccana*^a^	Morocco (Agadir)	M	6x	nuclear, plastid, mitochondrial	Olive genome project
*maroccana* 'Immouzzer S1'	Morocco (High Atlas)	M	6x	plastid	NCBI (NC_015623)
*cerasiformis*^a^	Portugal (Madeira)	M	4x	nuclear, plastid, mitochondrial	Olive genome project
*guanchica*^a^	Spain (Tenerife)	M	2x	nuclear, plastid, mitochondrial	Olive genome project
*guanchica* ‘La Gomera 10’	Spain	M-g1	2x	plastid	NCBI (MG255764)
*guanchica*-w6	Spain (Tenerife)	M	2x	nuclear, plastid, mitochondrial	NCBI (SRR9860513)
*guanchica*-w5	Spain (Gran Canaria)	M	2x	nuclear, plastid, mitochondrial	NCBI (SRR9860515)
*laperrinei*^a^	Sahara	E1.1	2x	nuclear, plastid, mitochondrial	Olive genome project
*laperrinei* ‘Adjelella 10’	Algeria	E1-l1	2x	plastid	NCBI (MG255765)
*cuspidata*-R	Reunion island	A	2x	nuclear, plastid, mitochondrial	ENA (PRJEB35540)
*cuspidata*-S^a^	South Africa	A	2x	nuclear, plastid, mitochondrial	Olive genome project
*cuspidata*-I^a^	Iran	C1	2x	nuclear, plastid, mitochondrial	Olive genome project
*cuspidata* 'Almihwit 5.1'	Yemen	C2	2x	plastid	NCBI (FN996943)
*cuspidata* 'Guanghzou 1'	China	C1	2x	plastid	NCBI (FN996944)
*cuspidata* 'Maui 1'	USA (Hawaii-Maui)	A	2x	plastid	NCBI (NC_015604)
*cuspidata* ‘Menagesha Forest 14’	Ethiopia	C2	2x	plastid	NCBI (MG255760)

^a^Samples sequenced in this project

To study structural changes within *O. europaea*, we focused on small insertions/deletions (indels). We identified a total of 1,504,957 indel positions that have sizes ranging from 1 to 313 nucleotides. Interestingly, the two polyploid individuals (subspp. *laperrinei* and *maroccana*) have the highest number of total indels and large indels (> 100 nucleotides) (Additional file 1: Table S1). When we searched for indels present in genic regions, we found 424,944 positions affecting genes. The number of genes affected by indels are similar for all individuals, with an average of 25,627 genes (Additional file 1: Table S2). GO term functional enrichment analysis shows enrichment for nucleus and cytoplasm cellular component, microtubule-based movement biological process, and different molecular functions, including microtubule motor activity, microtubule binding, phosphatase activity, palmitoyltransferase activity, ATP binding, and threonine-type endopeptidase activity (Additional file 1: Table S2). Interestingly, palmitoyltransferase activity is a key enzyme of sphingolipid metabolism [12], which has been shown to play an important role during olive fruit development and ripening [13, 14].

### Patterns of allelic representation in heterozygous positions

**Fig. 2:**
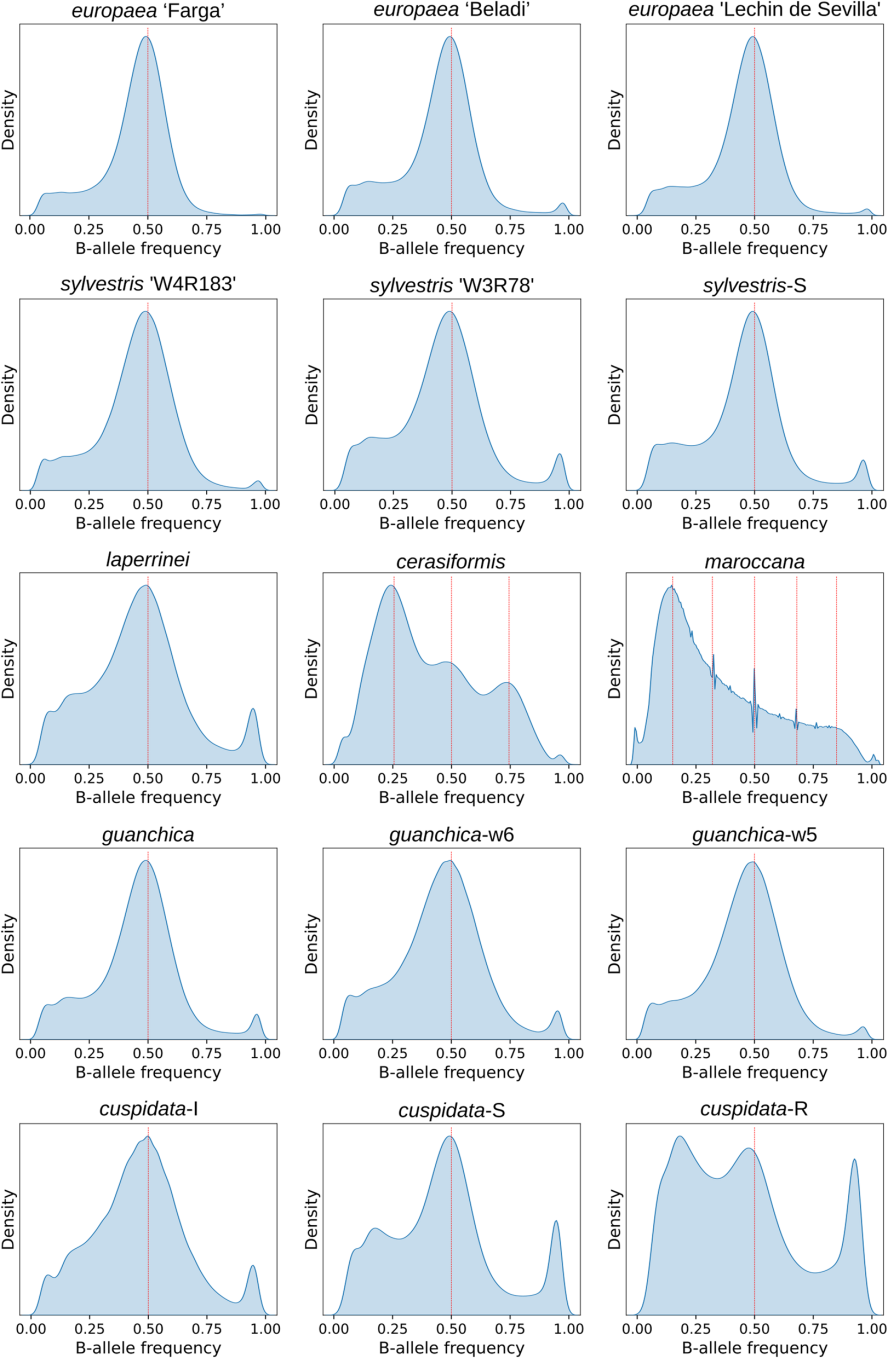
Density plot for the relative coverage of alternative alleles in heterozygous sites per individual. For all cases, we only plotted data corresponding to the 23 pseudo-chromosomes. Red line indicates the position of the peak consistent with the ploidy

To assess the ploidy of each sequenced individual we plotted the relative coverage of alternative alleles in heterozygous sites (see Methods). In these plots (Fig. 2) polyploid subspecies show allele frequency peaks to a certain extent consistent with their polyploid level: hexaploid (five peaks at 0.17, 0.33, 0.50, 0.67, 0.83) in subsp. *maroccana* and tetraploid (three peaks at 0.25, 0.50, 0.75) in subsp. *cerasiformis* [9]. For the diploid subspecies, we observed a prominent peak at the expected frequency of 0.50. In addition, a second peak, much broader and of lower height, was observed around frequencies between 0.05 to 0.25. As this secondary peak also appears in the reference, it may represent segmental duplications, aneuploidies, or repetitive regions that may be collapsed in the reference assembly. Differences in the height of the secondary peak could represent variations in the amount of such repetitive regions in other individuals. Interestingly, the subsp. *cuspidata* shows different patterns for the three individuals with *cuspidata*-I from Iran being diploid as previously reported [15], and the other two individuals (*cuspidata*-R and *cuspidata*-S) from Reunion Island and South Africa showing a higher secondary peak, which could reflect differences in genome size previously observed in this subspecies [9].

In order to determine whether repetitive regions are responsible for the secondary peak, we selected all the positions with values ranging from 0.05 to 0.25 (P25) and analysed if they fall inside repetitive regions or genes. Interestingly, an average of 58% of such positions falls in repetitive regions, while 21% falls in genes (Additional file 1: Table S3). The individuals with more P25 positions (> 60%) in repetitive regions are *cuspidata*-S (61%), *cuspidata*-R (63%), *sylvestris*-S (61%), and *sylvestris* ‘W3R78’ (63%), while the polyploid individuals show the lowest values (*maroccana*—44% and *cerasiformis*—46%). For the *cuspidata* subspecies, we observe that the individual that does not have a clear secondary peak, *cuspidata*-I, has the lowest percentage (57%) of P25 positions falling in repetitive regions. Additionally, when we performed a GO term enrichment analysis of the genes that contain the P25 positions, we found that ATP binding is enriched in the three *cuspidata* individuals, *laperrinei*, and *sylvestris* 'W3R78' (Additional file 1: Table S3). Interestingly, ATP-binding cassette transporters in plants are a very large and diverse protein family and their number can vary even within the same species [16–18]. Altogether, these results show evidence that this secondary peak may be a product of repetitive regions and segmental duplications containing genes.

### Genetic diversity in the *O. europaea* complex

Interestingly, small populations (such as subsp. *laperrinei*) and populations in oceanic islands (subsp. *guanchica*) are associated with decreased levels of genetic diversity [19, 20]. To test this, we calculated the genetic diversity of 4 groups of the *O. europaea* complex (Table 2). For the subsp. *europaea*, we analysed the two varieties independently and observed a slightly higher genetic diversity in var. *sylvestris*, which is in agreement with previous studies [10, 11, 21]. When we compare the three subspecies we can observe that subsp. *guanchica* has the highest genetic diversity (Nei, H = 2.8 × 10^–3^), followed by subsp. *cuspidata* (Nei, H = 2.6 × 10^–3^). Interestingly, subsp. *guanchica* is endemic to the Canary Islands, while the other two subspecies have a broad distribution, with subsp. *europaea*, in the Mediterranean Basin; and subsp. *cuspidata,* from South Africa to South Egypt and from Arabia to North India and South-West China. These results suggest that subsp. *guanchica* may originate from an ancient larger population with a broader ancestral distribution and was able to maintain their genetic diversity for a very long period.

**Table 2:** Nei’s gene diversity (H) of the subsp. *europaea* var. *europaea*, subsp. *europaea* var. *sylvestris*, subsp. *guanchica*, and subsp. *cuspidata*

*Olea europaea* subsp.	H
*europaea* var. *europaea*	2.31E-03
*europaea* var. *sylvestris*	2.33E-03
*guanchica*	2.75E-03
*cuspidata*	2.56E-03

### Analysis of heterozygous SNVs

**Fig. 3:**
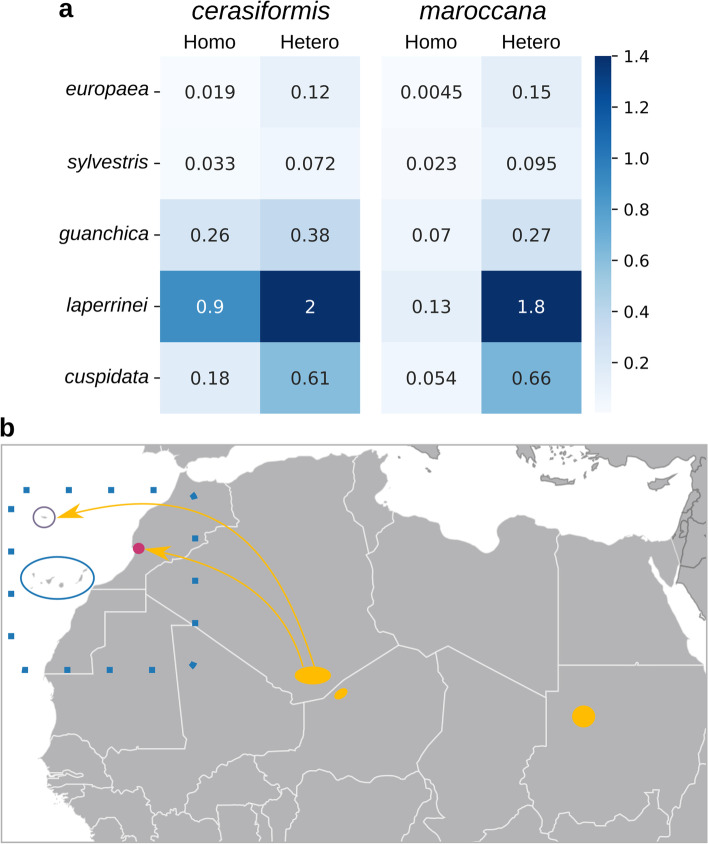
The SNV similarity between diploids and polyploids and the possible origin of the polyploids. **a** Heatmap showing the percentage of the SNV positions of the polyploids (*cerasiformis* and *maroccana*) shared with the diploid subspecies. Only the exclusive SNVs of the diploid subspecies were analysed. **b** Map depicting the possible origin of the polyploids. Approximate distribution of subsp. *laperrinei* in yellow and subsp. *maroccana* in pink. The empty circles show the distribution of subspp. *cerasiformis* (purple) and *guanchica* (blue). The dotted line rectangle represents the presumed ancestral distribution of subsp. *guanchica*. The distribution of the subspecies was taken from [22]

Polyploid subspecies show a high number of heterozygous SNVs (Fig. 1b, light striped colours), which may affect their phylogenetic positions because to reconstruct the nuclear phylogeny we only used homozygous positions (see Methods). To overcome this problem, we compared independently exclusive SNVs of each diploid subspecies (SNV positions only present in the analysed individual, polyploid individuals are excluded in this step) to those of the polyploids. Our results show that the polyploid subspecies share more exclusive homozygous SNVs with the subsp. *laperrinei* and *guanchica*, and more exclusive heterozygous SNVs with the subsp. *laperrinei*, *cuspidata*, and *guanchica* (Fig. 3a). These results support the idea that at the nuclear level, subsp. *laperrinei* is involved in the origin of the polyploids.

### Phylogenomic relationships in the *O. europaea* complex

**Fig. 4:**
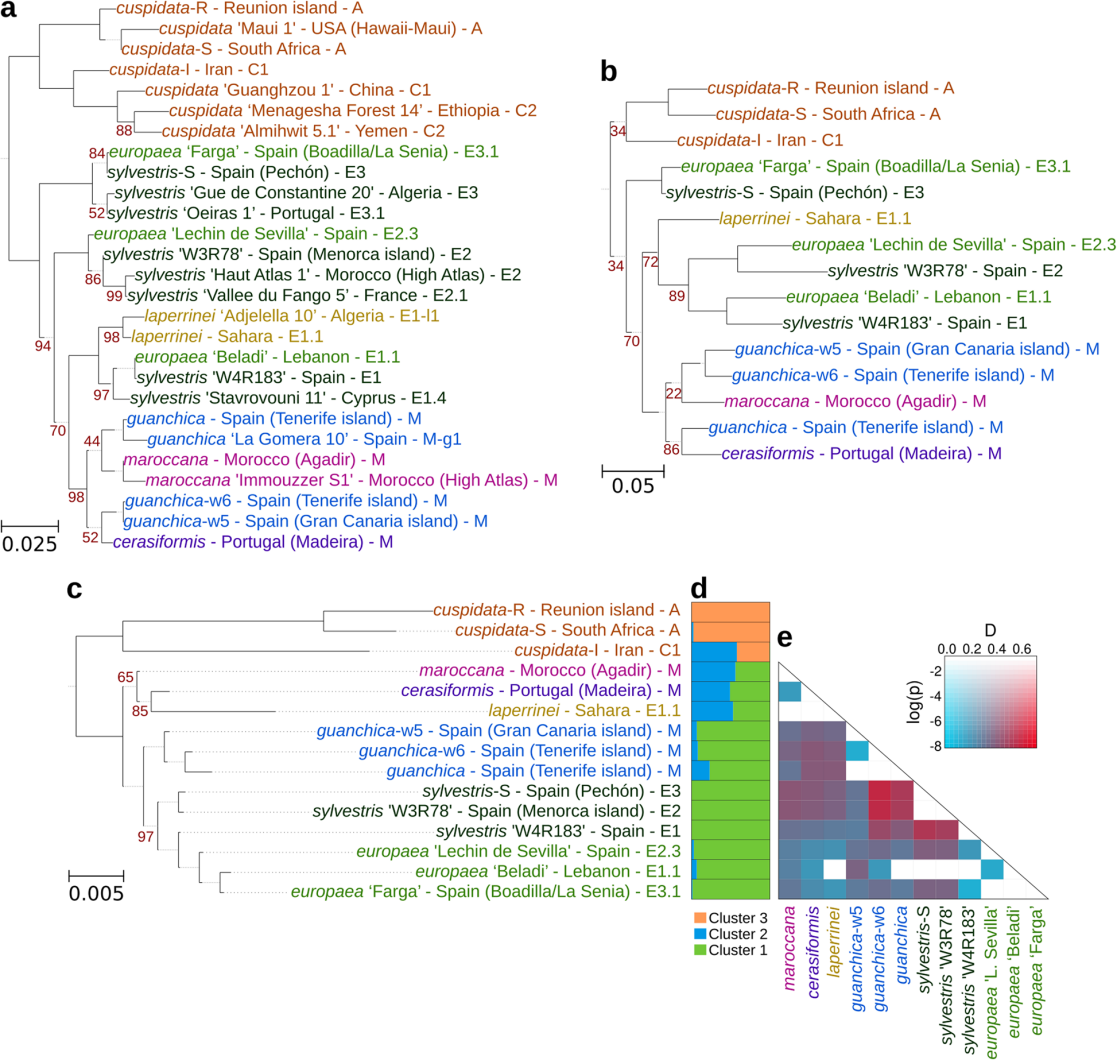
Evolutionary relationships among the individuals of the *O. europaea* complex. Maximum likelihood species tree for the plastid SNVs (**a**), mitochondrial SNVs (**b**), and the nuclear SNVs (**c)**. The geographical location of the accession and the plastid haplotype are indicated. Only bootstrap values below 100% are shown. **d** Bayesian clustering for the nuclear SNV data estimated in Structure v2.3 for the *O. europaea* complex. Structure bar plot shows the genetic clusters differentiated by colour. **e** Heatmap showing the D-statistic and its p value. Red colour indicates higher D-statistics, and more saturated colours indicate greater significance

To understand the evolutionary relationships among subspecies and sequenced individuals within the *O. europaea* complex, we ran a model-based genetic structure analysis using nuclear SNVs, and reconstructed phylogenetic trees using nuclear, mitochondrion, and plastid SNVs, separately (Fig. 4). Population structure analysis showed that the most likely number of ancestral genetic groups (k) among the *O. europaea* complex is k = 3 (Fig. 4d). These three clusters of genetic ancestry are differentially present among the various individuals (Fig. 4d, Additional file 1: Table S4). Only one of the groups (cluster 3) is exclusively present in one of the subspecies (*cuspidata*), supporting a deep divergence of this subspecies from the rest of the *Olea* complex. Another genetic cluster (1) is particularly found in subsp. *europaea* and *guanchica,* but it is also present, to a lesser proportion, in three other subspecies (*cerasiformis*, *laperrinei*, *maroccana*). The remaining genetic cluster (2) is more abundant in *cerasiformis*, *laperrinei*, *maroccana*, and *cuspidata* from Iran. This latter individual shows a higher proportion of the genetic cluster 2, which contrasts with the other *cuspidata* samples that are almost purely presenting cluster 3. Such mixed ancestries inferred from genetic data can be interpreted as arising from relatively recent admixture among multiple founder populations, but they can also result from shared ancestry already present before the divergence of the populations [23, 24].

Additionally, we reconstructed the phylogenomic relationships of the *O. europaea* complex using plastid, mitochondrial, and nuclear SNVs. The three genomes will tell us different evolutionary histories and help to unravel reticulation or incomplete lineage sorting [7, 25, 26]. Since in *O. europaea*, plastid and mitochondria are maternally inherited [27], they are expected to reflect similar evolutionary events. However, the nuclear genome can show a more complex history due to admixture and recombination between intermixing populations. Indeed, our results show that in the plastid and mitochondrial trees (Fig. 4a,b) the individuals group according to the previously described plastid types [7, 11]. However, when we compare the plastid and mitochondrial trees to the nuclear tree, we observe some incongruences (Fig. 4a,b,c), which is taken in other studies as evidence of hybridization or admixture processes [28–30].

Importantly, the subsp. *laperrinei* has a complex hybridization history, which suggests a likely involvement in the domestication of olive and the origin of polyploids. For instance, in the plastid tree, this subspecies appears closer to the subsp. *europaea* individuals that present eastern Mediterranean plastid type (E1) (Fig. 4a) and in the mitochondrial phylogeny, appears closer to the eastern and western type (E1, E2) (Fig. 4b), but in the nuclear tree, groups with the polyploid individuals (*cerasiformis* and *maroccana*) (Fig. 4c). These results indicate that hybridization occurred between *laperrinei* and *europaea,* which is in agreement with previous studies based on nuclear markers and plastid genomes [7, 31, 32]. In addition, these results support the possibility that this subspecies is involved in the origin of the polyploids.

The subsp. *guanchica* also shows a reticulate evolution. This subspecies appears closer to the subsp. *europaea* in the nuclear tree, while groups with the polyploid individuals in the plastid and mitochondrial trees. These results indicate that subsp. *guanchica* is also involved in the origin of the polyploids.

Not all the accessions of subsp. *europaea* cluster together in the plastid and mitochondrial trees (Fig. 4a,b). In the plastid tree, we can observe the subsp. *europaea* forms three different clusters in agreement with their plastid type, while in the mitochondrial tree two groups are formed. However, in the nuclear tree (Fig. 4c) all the individuals of the subsp. *europaea* group together, which indicates that they derived from a single ancestor(s), suggesting a monophyletic origin.

**Fig. 5:**
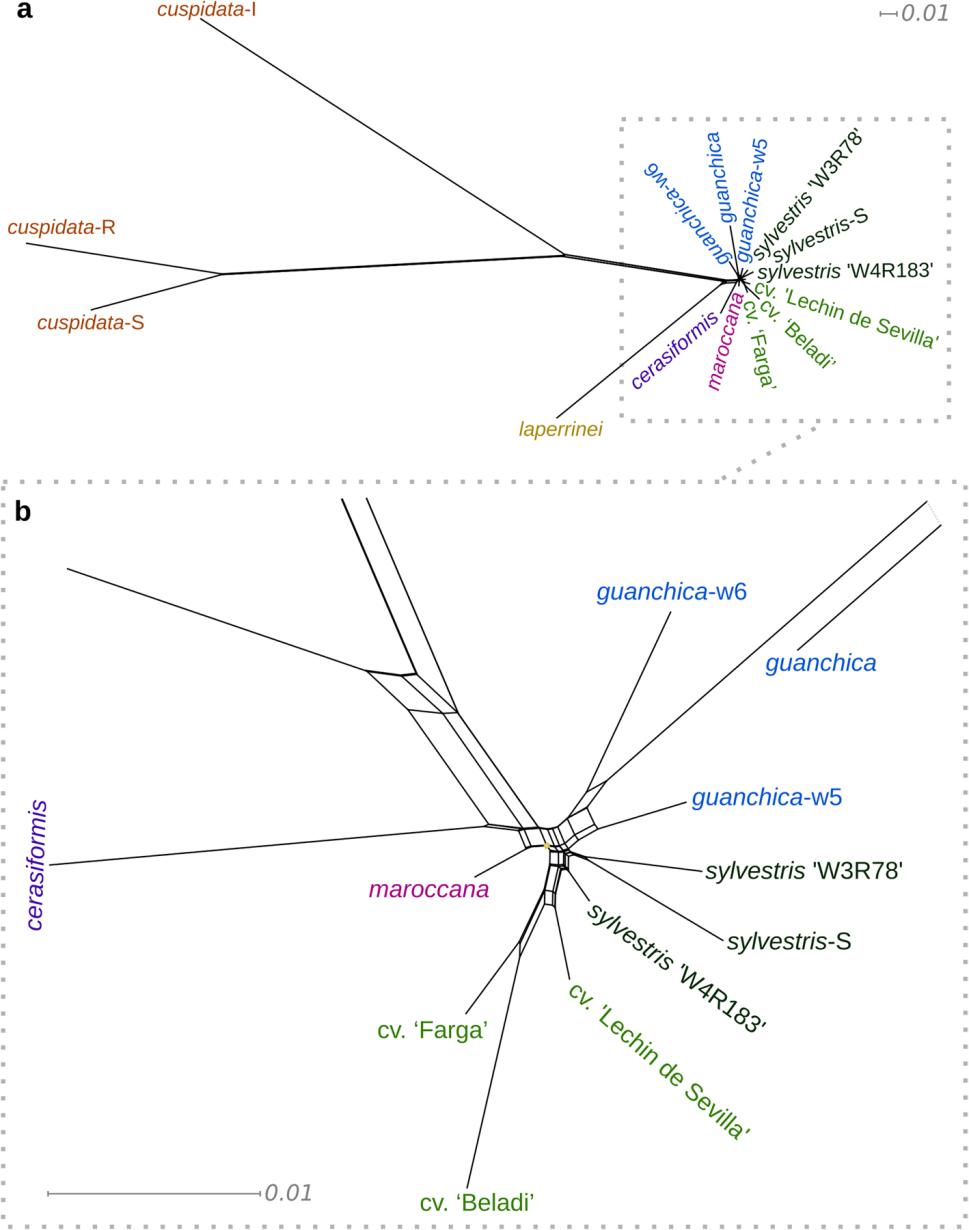
SplitsTree derived from nuclear SNVs. **a** Complete plot. **b** Zoom in on the region in the rectangle. The neighbor-net method is used here to explore data conflict and not to estimate phylogeny

Phylogenetic analyses, and previously proposed phylogenetic relationships, suggest the existence of rampant gene flow in the evolution of the *O. europaea* complex [10]. A split network tree (Fig. 5) reveals a heavily reticulated structure with conflicting phylogenetic signals affecting mostly the relationships among five subspecies, including the cultivated and wild olives, other than *cuspidata*. In this network, we notice that the most differentiated group is subsp. *cuspidata*, which is compatible with the proposed earliest divergence of this group [4, 8, 11]. The subspecies *europaea* and *guanchica* appear as monophyletic*.* Of note, the cultivars appear in an elongated, but also reticulated, part of the network likely reflecting a more profound sequence divergence due to domestication.

The reticulated network can result from hybridization (including introgression) or incomplete lineage sorting. To search for evidence of introgression among our samples, we ran the ABBA-BABA test [33] using the program Dsuite v0.5 r49 [34] for all trios of the phylogenetic tree (Fig. 4c). This analysis shows that introgression occurred between subsp. *guanchica* and var. *sylvestris* (Fig. 4e). This result highlights the contribution of *guanchica* to subsp. *europaea*.

## Discussion

### The early-most olive divergence

Genomic differences between the individuals of subsp. *cuspidata* are remarkable. As mentioned above, individuals from Reunion Island and South Africa (*cuspidata*-R and *cuspidata*-S) group together in a single genetic group, while *cuspidata*-I, from Iran, is a mixture of two clusters (Fig. 4d). Indeed, *cuspidata*-I shares more genetic ancestry with other subspecies from North Africa than with the other *cuspidata* from Africa (Additional file 1: Table S4). Moreover, in the nuclear and the organellar trees, the individuals of subspecies *cuspidata* form a monophyletic group (Fig. 4a,b,c). Interestingly, in all cases, the three individuals sequenced in this study are divided into two clades separated by a long branch: *cuspidata*-S and *cuspidata*-R on one side, and *cuspidata*-I on the other. Although our study includes a few individuals from subsp. *cuspidata*, our results show that African individuals highly differentiated from the Iranian individual. Differences between *cuspidata* from Africa and Asia were observed in previous studies based on organellar markers [7, 35–38]. These studies have shown that two geographically distant groups exhibit two different plastid types: “A” in Tropical and Southern Africa, and “C” in Southern Asia to Eastern Africa. Furthermore, in some regions of Iran, *cuspidata* populations occur close to cultivated olives suggesting the possibility of sporadic hybridization among them [39–41]. However, for our sequenced individual, introgression from cultivars can hardly explain the observed pattern of genetic ancestry, as cluster 2 from our structure analysis is only residually present in cultivars, whereas is common in other wild *O. europaea* subspecies from Africa. Interestingly, a recent analysis of plant reproductive structures in Asian and African *cuspidata* accessions has shown numerous differences at morpho-structural and functional levels, despite this variability was suggested to be due to different adaptability to the growth environment [42], may also indicate genetic differences. Considering the phylogenetic divergence between the Asian (Iran) individual and the other *cuspidata* accessions, one more taxon should be considered in the *Olea europaea* complex: subsp. *ferruginea*.

### Complex biogeographic scenario between the western Mediterranean and Macaronesian Islands

The present distribution of the *O. europaea* complex makes it an excellent model to study the colonisation of Macaronesian islands. Two subspecies, *guanchica* and *cerasiformis*, are endemic to the Macaronesian region and previous studies suggested two independent colonisation events to the Madeira and Canary Islands [43]. Our phylogenetic analysis shows that subsp. *guanchica* is genetically closely related to subsp. *europaea* (Fig. 4c). Moreover, despite being endemic to an oceanic archipelago (Canary Islands), its genetic diversity is higher than that within the other subspecies with wider distributions (subsp. *cuspidata*, subsp. *europaea*, Table 2). This high genetic diversity was also observed in other studies, which suggested that gene flow from subsp. *europaea* was the main driving factor [44]. We hypothesise that subsp. *guanchica* may originate at least once directly from the Mediterranean region. As the cultivated olive (subsp. *europaea*) has been planted across the Canary Islands since the arrival of Europeans, we cannot rule out gene flow with trees of the Canarias endemic subsp. *guanchica*. On the other hand, the colonisation of subsp. *cerasiformis* to a Macaronesian Island (Madeira) is a more complex scenario, which involves a polyploidization event. Recent studies highlight the importance of polyploidy in the evolution of island biodiversity [45]. However, to understand the role of polyploids in islands, we need to understand their origin. Up to date, four main hypotheses have been postulated to explain the origin of the polyploid subspecies *cerasiformis* and the other continental endemic polyploid subspecies (*maroccana*). For each polyploid, we can find the following hypotheses: i) *cerasiformis* = *guanchica* x *europaea* [9], *cerasiformis* = ancestor of *guanchica* x *guanchica* [44] ii) *cerasiformis* = *laperrinei* x *laperrinei* (triploid-bridge) [22, 46], iii) *maroccana* = *laperrinei* x *laperrinei* (triploid-bridge) [22, 46], and iv) *maroccana* = *cerasiformis* x *guanchica* [44]. All these hypotheses have in common subsp. *guanchica* and *laperrinei* as parental taxa, albeit none of them has postulated the combination of both subspecies for the origin of the two polyploids. Our results suggest that ancestral populations of subsp. *guanchica* acted as a maternal donor (Fig. 4a,b), while ancestral populations of subsp. *laperrinei* could be the paternal donor (Fig. 4c). However, the current geographical distribution of subsp. *laperrinei* (Saharan massifs) and subsp. *guanchica* (Canary Islands) makes difficult the explanation of the hybrid origin of the endemic polyploids (subsp. *cerasiformis*—Madeira and subsp. *maroccana*—Agadir Mountains) (Fig. 3b) [2, 22, 47]. Interestingly, for subsp. *laperrinei*, earlier studies have shown that its small and highly isolated populations are able to maintain high genetic diversity by the existence of relict trees persisting for a very long time associated with asexual multiplication [31]. Also, ancient gene flow between subsp. *laperrinei* and subsp. *europaea* during favourable periods was proposed [7, 31, 32]. Thus, we suggest that gene flow between subsp. *laperrinei* and subsp. *guanchica* may have occurred during similar periods. These findings show evidence that hybridizations with change in ploidy level from an ancestor shared with subsp. *laperrinei*, as paternal donor, and *guanchica*, as maternal donor, may have brought about the two polyploid subspecies (Fig. 3b): *cerasiformis* = *guanchica* x *laperrinei*, *maroccana* = *cerasiformis* x *laperrinei*, or *maroccana* = *cerasiformis* x *guanchica*. In sum, we hypothesise that an ancestral already polyploid subsp. *cerasiformis* is the one that colonised Madeira island.

Current geographic distributions of the subspecies do not help to reconstruct the two polyploidization events. Ancient colonisation of Macaronesian islands by the tetraploid Madeiran olive (subs. *cerasiformis*) and the diploid Canarian olive (subsp. *guanchica*) took place a long time ago (last two million years [9]), which makes difficult any reliable reconstruction. The same is true for the hexaploid olive of Agadir mountains (subsp. *maroccana*) and the diploid Saharan olive (subsp. *laperrinei*) in northern Africa where desertification has been broadly expanded in the last 50,000 years [7]. Massive extinctions may explain why major difficulties in conciliation between current olive distributions and parental lineages to generate the two olive polyploids. The hypothesis of significant olive extinction because of the expansion of the Sahara desert in different periods of the Pleistocene-Holocene is supported by biogeographic patterns such as those of the Rand flora that illustrates east–west plant disjunctions in Africa [48] and Canarian and southern Asian disjunctions in the case of the pine trees [49]. Patterns of significant olive extinction have also been supported by phylogenomics of whole genomes to better explain even more ancient polyploidization in *Olea* [10].

### Phylogenomic evidence for the origin of the olive tree

Many studies have pointed to the eastern Mediterranean Basin as the place where the first domestication event of olive occurred around 6,000 years ago [50, 51]. Recent studies have shown that this primary domestication was followed by numerous secondary events across Mediterranean countries, particularly involving genetic admixture with wild populations (var. *sylvestris*) from the western Mediterranean Basin [10]. However, few studies highlight the contribution of other subspecies to the evolution of olive. Our phylogenomic analysis shows that two subspecies are closely related to olive, subspp. *laperrinei* and *guanchica*. For the subsp. *laperrinei*, the organelles trees (Fig. 4a,b) show a closer relationship with the cultivars from the eastern Mediterranean Basin. This is in agreement with previous studies based on nuclear and plastid markers, which reveal historical hybridization between *laperrinei* and *europaea* during waning and waxing of African lineages as a result of climatic fluctuations during the Pleistocene [4, 8]. On the other hand, the nuclear phylogeny shows that the subsp. *europaea* is closely related to subsp. *guanchica*, which is considered a biogeographic connection between the Mediterranean and the Canarian olives. In sum, subspp. *laperrinei* and *guanchica* represent closely-related genetic pools to the Mediterranean olive (subsp. *europaea*) because of their genomic origin (ancestral gene flow with subsp. *laperrinei*, and colonisation of the Canary Islands bringing about subsp. *guanchica*).

## Conclusions

Altogether, based on the presented data a phylogeny-informed grouping of the nuclear genetic diversity present in seven subspecies of *Olea europaea* would result in the following clades: i) *ferruginea* (Asia), ii) *cuspidata* (Africa); iii) *laperrinei* (Sahara), iv) *cerasiformis* (Madeira), v) *maroccana* (Agadir Mountains, Morocco), vi) *guanchica* (Canary Islands), and vii) *europaea* (Mediterranean basin). The last one includes the domestication process, which included recurrent admixture between eastern Mediterranean domesticates and western Mediterranean wild populations.

The incongruences found between organellar and nuclear trees may reflect historical hybridization between main lineages of the *Olea europaea* complex. In particular, we propose a hybrid origin of the polyploid (allopolyploid) subspecies *cerasiformis* and *maroccana*. Previous results based on molecular phylogenetics agree with our phylogenomic hypothesis in which the diploid subsp. *laperrinei* and subsp. *guanchica* contributed to the formation of both allopolyploid subspecies.

It is important to note that northern Africa has suffered dramatic climatic changes determining the distribution of plant species in recent times. This can explain why some *Olea europaea* lineages are now restricted to narrow areas that may have been larger in the past. Indeed, given the current distribution of *laperrinei* (Saharan massifs) and *guanchica* (Canary Islands), and the presence of polyploids between these two clades in Madeira (*cerasiformis*) and Agadir Mountains (*maroccana*), we propose that the two clades encountered and formed hybrids in a presumably larger area spanning from the Atlas mountains to Madeira. As a result, the subspecies *maroccana* and *cerasiformis* appear to be allopolyploids that successfully adapted to the specific local conditions (narrow endemics).

In summary, our results show recurrent patterns of hybridization among most lineages of the *O. europaea* complex followed by lineage divergence and geographical isolation in agreement with previous molecular studies [4, 8, 36, 41]. Nevertheless, the use of 27 olive organellar and nuclear genomes gives us the opportunity to perform a more reliable reconstruction of geographic and phylogenetic origins of the Mediterranean olive tree (subsp. *europaea*) and relatives (six more subspecies).

## Methods

### Genome sequences

A total of 15 nuclear genome samples were analysed to represent the highest levels of genetic diversity in the *Olea europaea* complex. In particular, we sequenced the genome of five of the six subspecies of the *Olea europaea* complex [2]: two of *cuspidata*, and one of *cerasiformis*, *guanchica*, *maroccana*, and *laperrinei* (Table 1). DNA of each sample was extracted from leaf tissue as described in [6] and their genomes were sequenced using Illumina HiSeq 2000 paired-end technology to a sequencing depth ranging from 24 to 34 × as described in [6]. In addition to these six samples, we also included the publicly available data of six individuals of the subspecies *europaea*, one subsp. *cuspidata*, and two *guanchica* (see Table 1) [6, 10, 52]. In addition, we downloaded the plastid genome of twelve individuals belonging to the different subspecies of *O. europaea* from the NCBI database and one mitochondrial genome (Table 1).

### Detection of single nucleotide variants and small insertions/deletions

Sequence reads from each sample were mapped onto the nuclear, plastid, and mitochondrial reference genomes of *O. europaea* subsp. *europaea* cv. ‘Farga’ [10] using BWA 0.7.6a-r433 [53]. Single nucleotide variants (SNV) and small insertions/deletions (indels) were identified with GATK HaplotypeCaller v4.1.8.1 [54], setting ploidy according to the described ploidy level of the subspecies, i. e. hexaploid for *maroccana*, tetraploid for *cerasiformis*, and diploid for the other four subspecies. For SNVs we used the following filters: mapping quality (MQ > 40), quality by depth (QD > 2), Fisher strand bias (FS < 60), mapping quality rank-sum test (MQRankSum > -12.5), read pos rank-sum test (ReadPosRankSum > -8), strand odds ratio (SOR < 3), read depth of coverage (DP > = 10). We also removed both, sites with missing alleles and sites with more than two alleles. For indels we used the following filters: quality by depth (QD > 2), quality (QUAL > 30.0), Fisher strand bias (FS < 200). We also removed sites with missing alleles and sites supported only by one individual.

### Ploidy estimation using the relative coverage of alternative alleles in heterozygous sites

To assess the ploidy of each sequenced individual we used the nuclear SNV data and plotted the relative coverage of alternative alleles in heterozygous sites. We considered only the 23 pseudo-chromosomes and each individual was analysed independently. For each heterozygous position, we computed the relative coverage of alternative alleles by dividing the alternative allelic depth by the total depth at that position. For a diploid genome, we would expect a single peak around 0.50 at biallelic positions, for a tetraploid three peaks, around 0.25, 0.50, and 0.75, and for a hexaploid five peaks around 0.17, 0.33, 0.5, 0.67, 0.83. Finally, all plots were generated using the Seaborn [55] python package.

### Functional analysis of selected genomic regions

In order to determine whether the secondary peak observed at alternative allele frequencies of 0.25 is the product of differences in repetitive sequences among the *O. europaea* complex, we mapped those positions to the annotations of the repetitive regions and genes. We selected all the positions with frequencies ranging from 0.05 to 0.20 and we map their positions to the gff files of the repetitive regions and gene annotations of the Oe9 genome (https://denovo.cnag.cat/olive). Then, per each individual, we counted the number of positions that fall inside the regions of repetitive elements or genes. Finally, for the genes associated with those positions, we performed GO term enrichment analyses using FatiGO [56].

### Exclusive SNV analysis

To assess putative parental lineages of polyploid individuals, we assessed which diploid groups of the *O. europaea* complex share more SNVs with the polyploid individuals. The groups studied are the three individuals of subspecies *guanchica*, *cuspidata,* and *europaea* (var. *europaea* and *sylvestris*) and one individual of subsp. *laperrinei*. Exclusive SNVs were homozygous only or homozygous and heterozygous positions in the studied diploid group (i.e. A/A or A/A, A/T for subsp. *guanchica*), and homozygous only in the other diploid groups (i.e. T/T for var. *europaea*, var. *sylvestris*, subsp. *cuspidata*, subsp. *laperrinei*). Then, we searched for those positions in the polyploid individuals and if they shared at least one allele with the studied group, we counted that position as shared. Finally, we calculated the percentage that those shared SNVs represented from the total SNVs in the polyploids (subsp. *cerasiformis* homozygous SNVs = 2,502,578, subsp. *cerasiformis* heterozygous SNVs = 6,567,558, subsp. *maroccana* homozygous SNVs = 1,457,218, subsp. *maroccana* heterozygous SNVs = 9,844,351).

### Admixture mapping

Because of the large number of polymorphic positions in the nuclear genomes of the *O. europaea* complex, and computational limitations, we generated 10 subsets of 100,000 randomly chosen polymorphic positions without overlaps and analysed them in parallel. Then we identified population structure without a priori grouping assumptions, using the Structure software v2.3.4 [57]. Structure was run with 100,000 generations of ‘burn-in’ and 100,000 Markov chain Monte Carlo (MCMC) iterations after burn-in for increasing K values ranging from 1 to 6, considering independent alleles and admixture of individuals. Simulations were repeated 5 times for each value of K. The optimal number of genetic clusters was determined using the ΔK method [58] using the software Structure Harvester [59]. Finally, the optimal K value was visualised with DISTRUCT v1.1 [60].

### Phylogenetic analysis

Phylogenetic trees were reconstructed using SNVs data from nuclear, plastid, and mitochondrial genomes, separately. For the nuclear genome, we included only homozygous positions, excluding all positions with heterozygous SNVs. In all cases, the genome sequence of the sequenced individuals was obtained by replacing the SNV positions in the respective reference genome, resulting in a pseudo alignment of all the considered genomes. For the plastid genomes, we included additional sequences by aligning our genomes with the genomes available in the databases (Table 1) using MAFFT v7.305b [61]. All these alignments were trimmed using trimAl v1.4 [62] with options -st 1 and -complementary, to remove all the non-informative positions. The final alignment had 3,632,494 variable positions for the nuclear genome, 328 for the plastid genome, and 3,245 for the mitochondrial genome. Phylogenetic trees were reconstructed from these alignments using RAxML v8.2.12 [63] and the GTR model. Support values were calculated based on 100 bootstrap repetitions. Additionally, for the nuclear data, we reconstructed a phylogenetic network using SplitsTree4 v4.17.1 and the NeighborNet approach [64].

For the subspecies *guanchica*, *cuspidata* and *europaea* (var. *europaea* and var. *sylvestris*), we estimated the Nei’s gene diversity index [65] in order to assess the proportion of nucleotide variation in each subspecies.

### Analysis of introgression

The ABBA-BABA test [33] was used to search for introgression among the *O. europaea* samples. Dsuite 0.5 r49 [34] was employed to calculate the D-statistic from nuclear SNV data for all subsets of trios that were compatible with the previously reconstructed phylogenetic tree (see above) using subsp. *cuspidata* as outgroup. For multiple hypotheses testing, we applied a false discovery rate correction to the p-values [66]. Then a heatmap showing the D-statistic and its p-value was plotted for all pairs of individuals using the plot_d.rb script (https://github.com/millanek/tutorials/blob/master/analysis_of_introgression_with_snp_data/src/plot_d.rb).

## Supplementary Information

**Additional file 1: Table S1.** Number of small insertions/deletions (indels) per kilobase. The columns in order show the number of homozygous indels, heterozygous indels, total number of indels, large indels (>100 nucleotides), indels in genic regions. The *O. europaea* individuals are in rows. **Table S2.** Number of genes of each *O. europaea* individual containing indels and GO term enrichment analysis of those genes. **Table S3.** Analysis of the positions with relative coverage of alternative alleles in heterozygous sites values ranging from 0.05 to 0.25 (positions P25). The columns show: name of the individual, number of positions P25, number of P25 positions present in repetitive regions, percentage of P25 positions present in repetitive regions, number of P25 positions present in genes, percentage of P25 positions present in genes, number of genes associated with P25 positions, GO term enriched function of the genes associated with P25 positions with *p*-value < 0.01. **Table S4.** Admixture coefficient (Q) of each individual per cluster. This table was used to create Fig. 4d.

## Data Availability

All data generated or analysed during this study are included in this published article, its additional files and publicly available repositories. The sequencing data produced in this project have been deposited in the ENA (European Nucleotide Archive) under the accession number PRJNA841802 [67]. The other datasets analysed in this study are available in the NCBI and ENA databases, and the accession numbers are indicated in Table 1.

## Abbreviations

subsp.: Subspecies (singular)
subspp.: Subspecies (plural)
var.: Variety
ENA: European Nucleotide Archive
NCBI: National Center for Biotechnology Information
SNVs: Single nucleotide variants
indels: Insertions/Deletions
ATP: Adenosine triphosphate
GO: Gene Ontology
P25: SNV positions with relative coverage of alternative alleles ranging from 0.05 to 0.25
MQ: Mapping quality
QD: Quality by depth
FS: Fisher strand bias
MQRankSum: Mapping quality rank-sum test
ReadPosRankSum: Read pos rank-sum test
SOR: Strand odds ratio
DP: Depth of coverage
QUAL: Quality
MCMC: Markov chain Monte Carlo
GTR: General Time Reversible
